# FlyBase: establishing a Gene Group resource for *Drosophila melanogaster*

**DOI:** 10.1093/nar/gkv1046

**Published:** 2015-10-13

**Authors:** Helen Attrill, Kathleen Falls, Joshua L. Goodman, Gillian H. Millburn, Giulia Antonazzo, Alix J. Rey, Steven J. Marygold, the FlyBase consortium

**Affiliations:** 1 Department of Genetics, University of Cambridge, Downing Street, Cambridge, CB2 3EH, UK; 2 The Biological Laboratories, Harvard University, 16 Divinity Avenue, Cambridge, MA 02138, USA; 3 Department of Biology, Indiana University, Bloomington, IN 47405, USA

## Abstract

Many publications describe sets of genes or gene products that share a common biology. For example, genome-wide studies and phylogenetic analyses identify genes related in sequence; high-throughput genetic and molecular screens reveal functionally related gene products; and advanced proteomic methods can determine the subunit composition of multi-protein complexes. It is useful for such gene collections to be presented as discrete lists within the appropriate Model Organism Database (MOD) so that researchers can readily access these data alongside other relevant information. To this end, FlyBase (flybase.org), the MOD for *Drosophila melanogaster*, has established a ‘Gene Group’ resource: high-quality sets of genes derived from the published literature and organized into individual report pages. To facilitate further analyses, Gene Group Reports also include convenient download and analysis options, together with links to equivalent gene groups at other databases. This new resource will enable researchers with diverse backgrounds and interests to easily view and analyse acknowledged *D. melanogaster* gene sets and compare them with those of other species.

## INTRODUCTION

Given the wealth of post-genomic data, it should be straightforward to query a biological database and obtain a robust list of genes related by the shared attributes of their products, such as actins, protein kinases or subunits of the proteasome. However, this is often not the case. For evolutionary-related genes, BLAST (1) or domain-based searches may yield a good preliminary list, but without further analysis, particularly when inferring function from sequence, it is hard to distinguish false positives. Additionally, there may be false negatives because a gene may be somewhat atypical or fails to score above a given threshold. Gene Ontology (GO) annotations can be used to search for gene products that are related by common biological attributes, but annotation is not exhaustive and expressing features that pertain to sequence does not fall within its scope (2,3).

An alternative approach to finding related genes (at least in species with well-characterized genomes such as humans and model organisms) can be to search for gene symbols that share a common prefix. This can be effective for databases such as WormBase (4) or the HUGO Gene Nomenclature Committee (HGNC) (5) that assign unifying and systematic gene symbols to nematode and human genes, respectively, based on shared structures, functions or phenotypes. However, this strategy is not generally applicable to *Drosophila melanogaster* genes in FlyBase (6) as these are traditionally named on a gene-by-gene basis by the authors who first publish on the gene, often reflecting a specific mutant phenotype.

A third method for acquiring a set of related genes is to directly consult relevant research or review articles. The advantage of this approach is that the list is compiled directly from an expert and peer-reviewed source, and as such will be robust and clearly attributable. However, it can be time-consuming to seek out and then extract a set of genes from individual publications (or, often, their supplementary data) and lists obtained in this manner are inherently uncoupled from the relevant species database, meaning that the listed gene symbols/IDs may become stale over time.

Several databases have addressed these issues by providing explicit sets of related genes. These are generally either inter-species databases that are focused on a particular functional attribute (e.g. kinase.com (7), Ribosomal Protein Gene database (8)), or are organism-specific databases that include discrete lists of related genes (e.g. HGNC (9), The Arabidopsis Information Resource (TAIR) (10) or WormBase (4)). Additional utility and value is given to these latter cases as the gene sets are linked to many other types of data (expression data, phenotypes, GO annotations, etc.) housed within such databases.

FlyBase (flybase.org), the primary database of biological information about *Drosophila* (6), has now created a ‘Gene Group’ resource for *D. melanogaster*, enabling researchers to gain easy access to acknowledged sets of fly genes. FlyBase Gene Groups are reliable and of high-quality as they are manually curated from the primary literature, and also benefit from full integration with the associated data and analysis tools within the database. This resource was first launched in the FB2015_02 release (May 2015) of FlyBase, and to date (FB2015_04, September 2015), there are close to 300 discrete Gene Groups comprising over 2100 unique genes (≈15% of all protein-coding genes; Table 1). Herein, we summarize our curation strategy, describe the features of the new ‘Gene Group Report’ webpages, and show how Gene Group data can be queried, downloaded and further analysed.

**Table 1.** Summary of Gene Group data in FlyBase (FB2015_04)

Gene Groups (total)	278
- terminal (gene-populated) Gene Groups	221
Genes in Gene Groups	2,143
- as a proportion of genome-localized genes	12.1%
- as a proportion of protein-coding genes	15.4%
Links to external sites and databases (total)	206
- HGNC	95
- WormBase	79
- TAIR	20
- Other	12

## CURATION STRATEGY

FlyBase Gene Groups are currently limited to well-defined, easily delimited groupings such as evolutionary-related gene families (e.g. actins, heterochromatin protein 1 family), subunits of macromolecular complexes (e.g. ribosome, SAGA complex) and other sets of genes whose products share a common molecular function (e.g. phosphatases, GPCRs, ubiquitin ligases).

**Figure 1.**
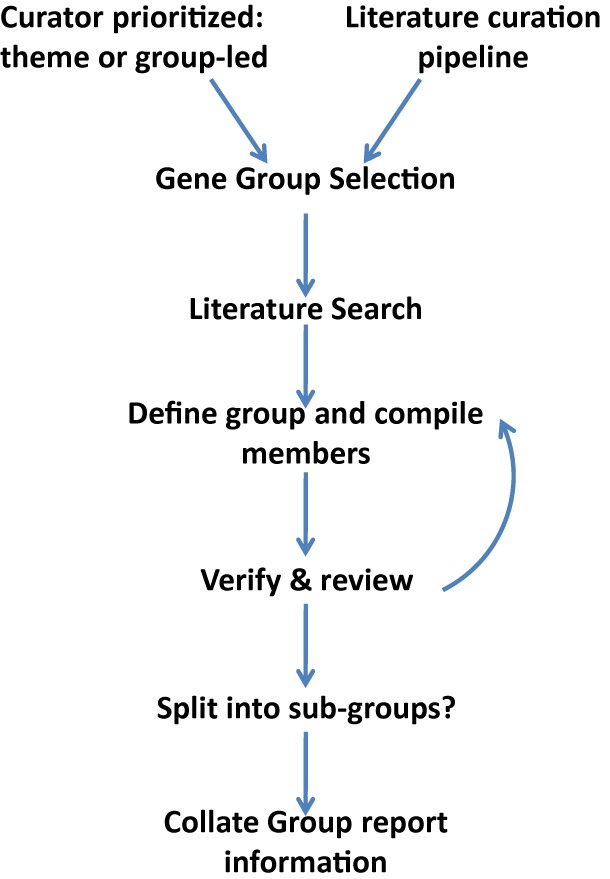
Gene Group Curation Strategy. A generalized scheme detailing the workflow for producing Gene Groups in FlyBase. See text for details.

A Gene Group is selected for curation through one of two main mechanisms (Figure 1): (i) a publication is ‘flagged’ as containing relevant data through the regular FlyBase literature curation pipeline (11); or (ii) a curator selects an important and established gene set (e.g. GPCRs, protein kinases, ribosomal subunits) or field (e.g. protein ubiquitination, chromatin modification, intracellular transport), sometimes using other resources for guidance (9,12). In either case, a systematic search of the literature is then conducted to identify all major publications that describe the group—in this way genes identified in multiple sources receive additional support for membership of a group, whereas genes that differ between sources can be investigated further. The emerging membership of a group is then crosschecked against current GO annotations, protein domain information and other FlyBase data using our query tools. Any additional genes thus identified are examined to determine whether they should be added to the group and, if so, to find supporting literature. As the Gene Group is compiled, a free-text description of the group is gradually refined and any ‘edge-cases’ where the inclusion/exclusion of a specific gene is unclear or debated are noted.

A search is also conducted for web-based resources that are relevant to the group, such as equivalent gene sets at other species databases. These external resources are recorded for inclusion in the final Gene Group Report (see below) and, in some cases, may contribute to the compilation of the FlyBase list.

Some Gene Groups are split into subgroups, creating hierarchical parent and child relationships between groups. These may represent formal classifications of gene families (e.g. GPCR > CLASS B GPCRs > SUBFAMILY B2), or more functional distinctions (e.g. the ACETYLCHOLINE RECEPTORS Gene Group is divided into MUSCARINIC and NICOTINIC subgroups), and largely reflect the organization of the groups in the primary sources. Rarely, a Gene Group will validly have more than one parent group (e.g. MUSCARINC ACETYLCHOLINE RECEPTORS is a subgroup of both AMINE RECEPTORS and ACETYLCHOLINE RECEPTORS Gene Groups). In each case, member genes are associated only with the terminal child group(s) within the hierarchy, with the membership of parental group(s) being inferred through their relationship with the child groups. Non-hierarchical, but still biologically relevant relationships between groups, such as ligands and receptors (e.g. WNTs and FRIZZLED-TYPE RECEPTORS) or enzymes with opposing actions (e.g. UBIQUITIN LIGASES and DEUBIQUITINASES) are also recorded.

The last step is to finalize the metadata describing the list of genes, which includes providing a name and symbol to use for the Gene Group in FlyBase, together with any commonly used synonyms. If an accepted abbreviation for the given Gene Group exists, or the same prefix is used in all or most of the symbols of the member genes, then this will be used as the Gene Group symbol; if not, FlyBase curators assign a suitably terse symbol.

## FINDING GENE GROUPS

**Figure 2.**
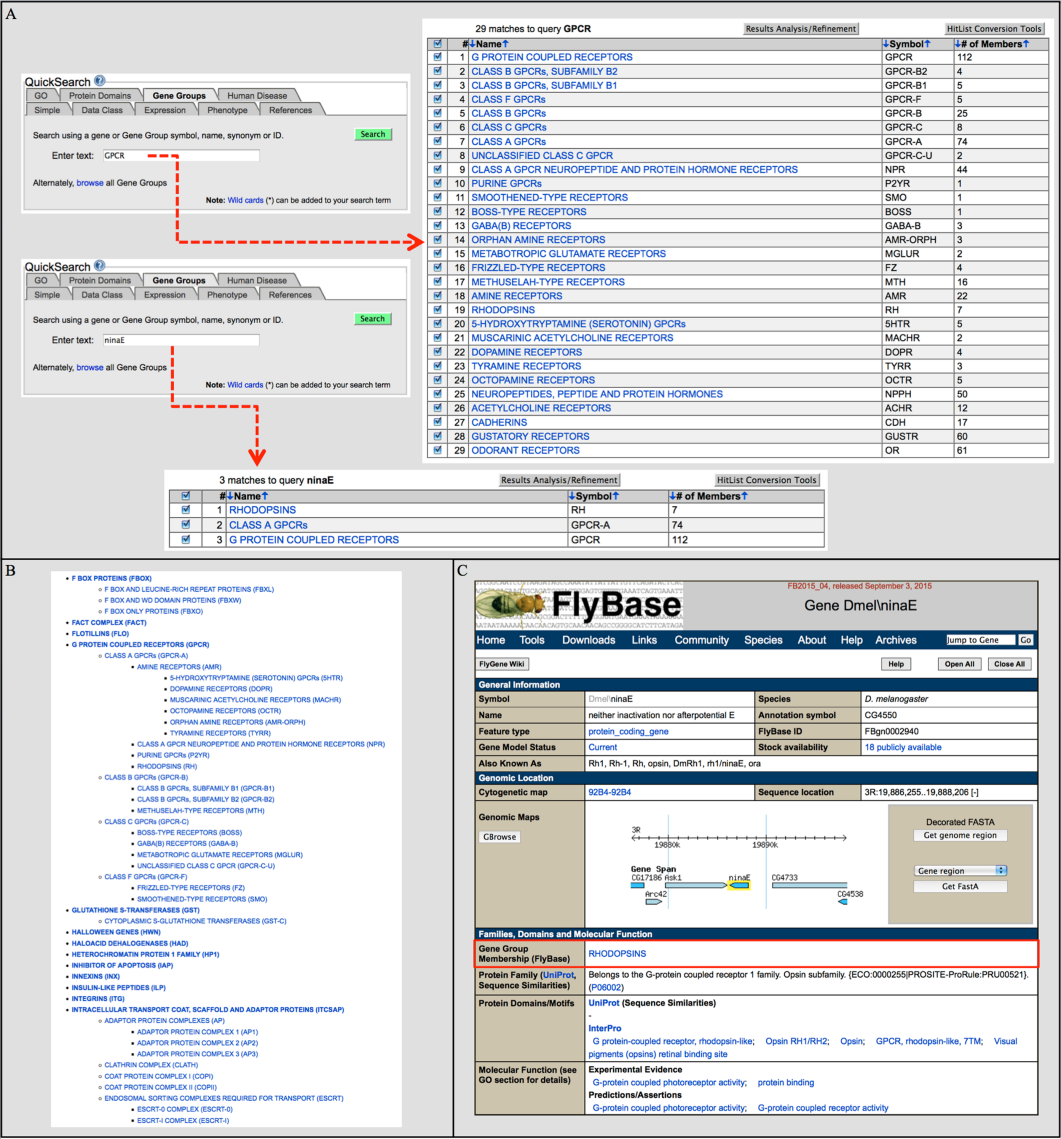
Finding Gene Groups. (**A**) The QuickSearch ‘Gene Groups’ tab can be searched using a symbol/name of a group (a search for ‘GPCR’ returns 29 hits that contain ‘GPCR’ in the text of the Report) or a member gene (a search for ‘*ninaE*’ yields 3 hits from the rhodopsin GPCR hierarchy). (**B**) Clicking on the ‘browse’ link in the ‘Gene Groups’ tab of QuickSearch takes the user to an alphabetical, nested list of all FlyBase Gene Groups. The section shown focuses on the GPCR hierarchy. (**C**) Gene Group membership is indicated within the ‘Gene Group Membership (FlyBase)’ field (red box) of the ‘Families, Domains and Molecular Function’ section of the Gene Report. In this example, the upper part of the *ninaE* Gene Report page is shown and the RHODOPSINS Gene Group is displayed.

There are three main ways to access Gene Groups in FlyBase. First, the QuickSearch tool on the homepage has a dedicated ‘Gene Groups’ tab (Figure 2A). This can be used to search for Gene Groups either by group name/symbol/ID or by the symbol/name of a member gene. For both options, Gene Groups that match the query will be displayed in a FlyBase HitList (unless a single hit is obtained). Alternatively, entering a search term in the Simple Search tab of QuickSearch yields results across all FlyBase data classes, including Gene Groups.

A second option is to browse all the available Gene Groups by clicking on the ‘browse’ link within the Gene Groups tab of QuickSearch. This is useful to see an overview of the current groups or to employ a ‘find in page’ approach. The list of Gene Groups is presented in a hierarchical structure, with subgroups nested under parent groups, and all group names are hyperlinked to their corresponding report pages (Figure 2B).

A third route to Gene Groups is via individual Gene Report pages of the member genes. Any Gene Group to which a gene belongs is shown as a hyperlink in the ‘Gene Group Membership (FlyBase)’ field (Figure 2C). This field is within the ‘Families, Domains and Molecular Function’ section of the Gene Report, allowing easy comparison with imported data from UniProt and InterPro classifications. Additionally, the textual description of any relevant Gene Group is repeated in the ‘Summaries’ section of the Gene Report, and a link is also provided here (not shown in Figure 2C).

FlyBase Gene Groups are also accessible via the HGNC website, where there are reciprocal links from human Gene Family pages back to any equivalent *D. melanogaster* Gene Groups on FlyBase. This portal will be especially useful to non-*Drosophila* researchers who are primarily using the HGNC site to search human gene data and are also interested in the orthologous set of fly genes, but might not visit FlyBase directly.

## GENE GROUP REPORTS

**Figure 3.**
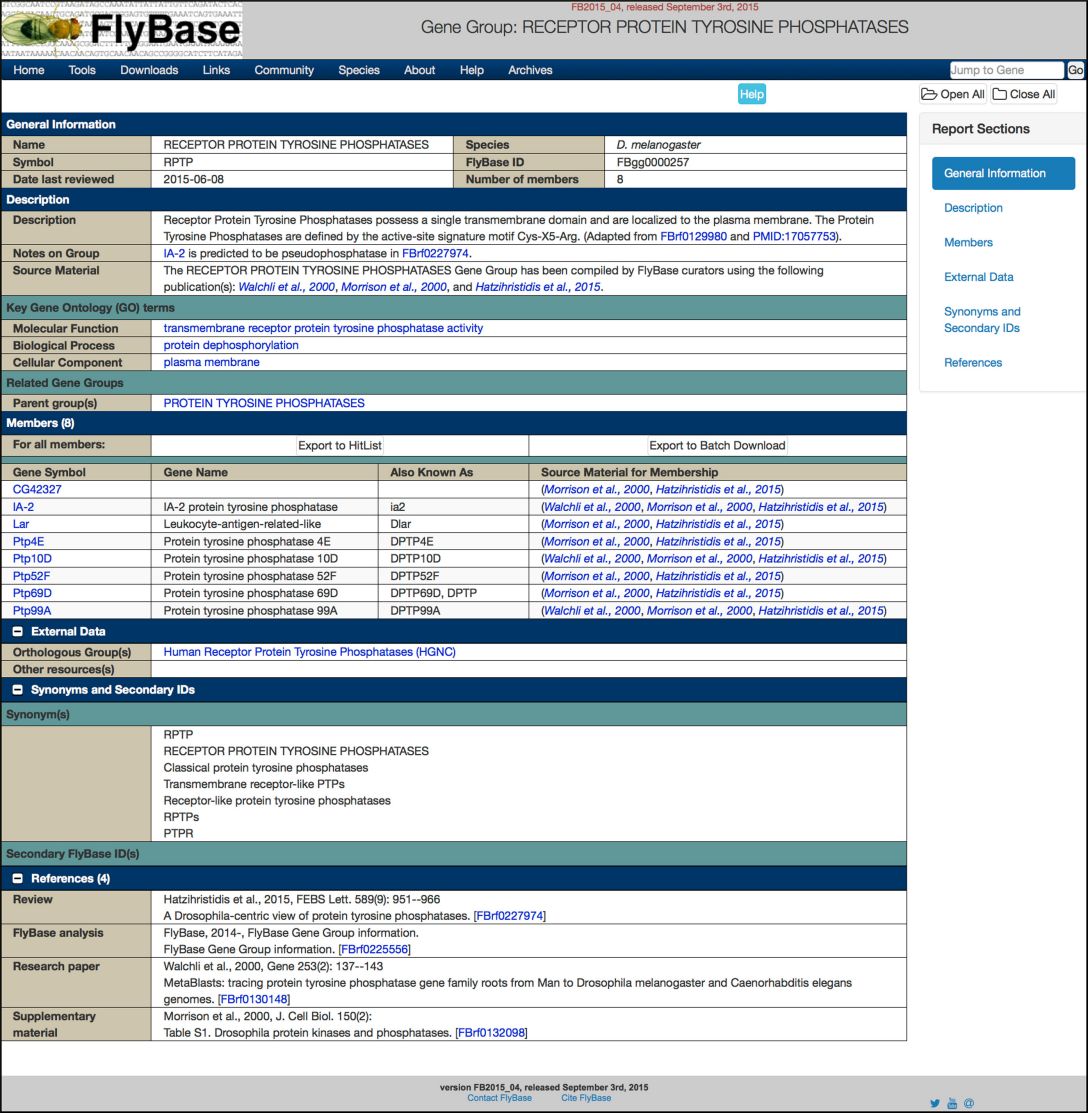
The Gene Group Report. The example shown is for the RECEPTOR PROTEIN TYROSINE PHOSPHATASES group. This group has one immediate parent group (PROTEIN TYROSINE PHOSPHATASES), shown in the ‘Related Gene Groups’ subsection. See text for details.

Gene Group data in FlyBase are presented in the form of Gene Group Reports (Figure 3), similar in style to that of other FlyBase Report pages with data organized into sections. For Gene Groups that are arranged into hierarchies, parent groups display all subgroups and their member genes, while each subgroup also has its own dedicated page. Importantly, the content and layout of the Gene Group Reports was finalized based on feedback from our recently formed ‘FlyBase Community Advisory Group’ (FCAG)—a group of >550 representatives from the research community (see the FCAG link on the ‘Community’ menu of the navigation bar of any FlyBase page). An example Gene Group Report is shown in Figure 3 and a brief description of each section is given below—additional help is available by clicking the ‘Help’ button at the top of the Report.

### General information

This section contains the identifiers for the Gene Group: the name, symbol and FlyBase Gene Group ID (FBgg). Note that FlyBase Gene Group names and symbols use all uppercase letters—this is to emphasize the status of Gene Groups as collections of genes and to help users distinguish Gene Groups from the genes themselves as they are encountered across the website. The General Information section also displays the number of genes within the group and the date that the group was last reviewed by FlyBase.

### Description

This section is split into three subsections. The first summarizes what the Gene Group is and how it has been compiled. The ‘Description’ field is a textual summary of the properties and attributes of the group written in a largely species-independent manner by FlyBase curators, adapted from the stated reference(s). The ‘Notes on Group’ field adds clarity as to how the FlyBase Gene Group has been compiled and/or highlights particular issues with its composition. For example, a justification for why certain genes have been included or excluded from a group, or an explanation of the nomenclature or classification system used. The ‘Source Material’ field serves to clearly state that the group has been compiled by FlyBase curators rather than via an automated pipeline, and provides a brief, but prominent, summary of the primary publications used.

The second subsection, ‘Key Gene Ontology (GO) terms’, displays the GO terms that best describe the properties expected of the Gene Group members—all or most of the individual Group members are annotated with these GO terms or one of their more specific child terms. While these terms, either alone or in combination, can rarely be used to completely define the group, they provide familiar and succinct ‘handles’ that can be used to form a quick impression of a group. They may also be helpful for comparing similar groups in other organisms or databases. The given GO terms are hyperlinked to their respective ‘Term Report’ page in FlyBase, where a definition of the term and other related information can be found (13).

Finally, the ‘Related Gene Groups’ subsection shows any parent and/or child (component) groups, hyperlinked to their own Report pages. Groups that are related in some other biologically relevant manner (ligands-receptors, etc.) are shown as ‘Other Related groups’.

### Members

The ‘Members’ table is the primary focus of the Gene Group Report and lists all the member genes of the given group, organized into subgroups where appropriate. The first two columns in the table are the gene symbol, hyperlinked to the corresponding Gene Report, and the gene name. The third column, ‘Also Known As’, is a computed field that displays the most frequently used symbol synonyms of the gene, and is particularly useful where the official FlyBase symbol does not follow a systematic nomenclature (e.g. it is based on a specific mutant phenotype rather than the function of its product).

Feedback from the FCAG indicated a strong preference for clearly displaying the sources used for compiling FlyBase Gene Groups, down to the level of individual gene membership. Thus, the final column in the Members table is labeled ‘Source Material for Membership’ and gives the publication(s) stating that a particular gene is a member of the given group, hyperlinked to the corresponding Reference Report for convenience. In addition this column allows the user to judge the weight of evidence supporting each gene's inclusion in the group and can highlight newly described members or those that are less-well characterized.

The top of the ‘Members’ section includes buttons for exporting gene members to other tools for further analysis or download—these features are described below.

### External data

The External Data section contains links to other databases and websites relevant to the given group. The links between *D. melanogaster* Gene Groups and equivalent groups at the HGNC, TAIR and WormBase databases are given in the ‘Orthologous Group(s)’ subsection. Links to other specialist web resources (e.g. kinase.com for the PROTEIN KINASES group) are displayed in the ‘Other resources(s)’ field. To date, there are over 200 unique links from FlyBase Gene Group pages to external data sites (Table 1).

### Synonyms and secondary IDs

This section simply states any alternative symbols and/or names that are commonly used to refer to the given group, either in *Drosophila* or in the wider field. Any FlyBase IDs previously associated with the group are also listed. The main function of this section is to provide a list of synonyms that users might use when searching for the group in FlyBase.

### References

This section is common to all Report pages and is organized in the standard FlyBase format (14). It lists the full citations of all References used to compile the Gene Group, as cited elsewhere in the Report.

## ANALYSIS AND DOWNLOAD OPTIONS

To extend the utility of Gene Groups and aid further analysis, two export options are provided at the top of the Members table in the Gene Group Report (Figure 3). By clicking ‘Export to HitList’, all genes within the Gene Group are exported to a standard FlyBase HitList (13). Here, the gene list can be refined, analysed, converted (to a related data type, such as alleles of those genes) or exported to a range of FlyBase tools for further processing. Alternatively, by selecting ‘Export to Batch Download’, the user can download specified data related to each member gene in various formats (13). For example, gene, mRNA or protein sequences in FASTA format, or a mix-and-match of the data included in the Gene Report (expression data, GO annotations, orthologs, Interpro domains, etc.) as an HTML table or tsv file. For Gene Groups that have a hierarchical structure (e.g. PROTEIN KINASES > TYROSINE KINASES > RECEPTOR TYROSINE KINASES), the exported data corresponds to the specific parent/child group page being viewed when the ‘Export’ button is clicked.

To facilitate bulk processing, FlyBase Gene Group data are also available as two precomputed files on the FlyBase FTP site or via the ‘Downloads’ menu of the navigation bar. The first file includes the symbol, name and ID of every group, any parent/child relationships between groups, and the symbol and ID of all member genes. The second file lists just the groups themselves together with any corresponding HGNC ‘gene family’ IDs.

## PERSPECTIVE

FlyBase is a comprehensive and complex database of *Drosophila* biology that serves a variety of users. The Gene Group resource for *D. melanogaster* described herein represents an important step toward data integration, providing an intuitive portal for researchers of all backgrounds to find sets of related fly genes. Moreover, by embedding this resource within FlyBase, the value of these groups is significantly enhanced beyond that of a simple list of genes for at least three reasons. First, it directly connects the member genes to the huge body of other data captured and hosted by FlyBase, thereby linking them to updates of the genome or nomenclature, and allowing interrogation and analysis of associated data. Second, the ‘group approach’ to curation feeds back to improve data quality in FlyBase: GO annotations and nomenclature of individual genes are reviewed for accuracy and consistency, and any unlocalized (‘non-CG’) genes that are identified during compilation of a group are merged with localized genes where possible. Third, the provision of links to equivalent gene sets hosted elsewhere facilitates navigation between different Model Organism Databases and other relevant resources.

FlyBase Gene Groups are intended to provide tight, discrete sets of genes, based on the published literature. For compiling broader lists of target genes for large-scale screens, particularly related to biological processes, users may wish to consult GLAD (Gene List Annotation for *Drosophila*), compiled by the *Drosophila* RNAi Screening Center (12). In general, this resource has focused on larger sets of genes with broader selection criteria. For example, the GLAD ‘Autophagy-related’ gene list contains 208 *Drosophila* orthologs of genes identified in a comprehensive proteomic analysis of the human autophagy interaction network (15), whereas the corresponding group in FlyBase is limited to 22 unique genes: the set of 20 evolutionarily conserved core ATG genes (the AUTOPHAGY-RELATED GENES group), plus two additional genes whose products contribute to one of the three characterized AUTOPHAGY-RELATED COMPLEXES (16,17).

FlyBase will continue to expand its Gene Group resource by focusing on established, well-characterized sets. Additionally, groups of topical interest will be targeted and authors are encouraged to indicate if new publications contain gene group data when using our ‘Fast Track Your Paper’ tool, accessible via the button on the homepage (18). Users may also suggest other groups or revisions to existing groups by clicking the ‘Contact FlyBase’ link in the footer of any FlyBase page.
